# Postoperative shoulder imbalance in Lenke Type 1A adolescent idiopathic scoliosis and related factors

**DOI:** 10.1186/1471-2474-15-366

**Published:** 2014-11-05

**Authors:** Morio Matsumoto, Kota Watanabe, Noriaki Kawakami, Taichi Tsuji, Koki Uno, Teppei Suzuki, Manabu Ito, Haruhisa Yanagida, Shohei Minami, Tsutomu Akazawa

**Affiliations:** Department of Orthopaedic Surgery, Keio University, Shinanomachi 35, Shinjuku-ku, Tokyo #160-8582 Japan; Department of Orthopaedic Surgery, Meijo Hospital, Nagoya, Japan; Department of Orthopaedic Surgery, National Hospital Organization, Kobe Medical Center, Kobe, Japan; Department of Orthopaedic Surgery, National Hospital Organization, Hokkaido, Medical Center, Sapporo, Japan; Department of Orthopaedic Surgery, Fukuoka Children’s Hospital, Fukuoka, Japan; Department of Orthopaedic Surgery, Seirei Sakura Citizen Hospital, Sakura, Japan

**Keywords:** Lenke type 1A, Postoperative shoulder imbalance, Adolescent idiopathic scoliosis

## Abstract

**Backgrounds:**

The purpose of this study was to investigate the occurrence and factors associated with postoperative shoulder imbalance (PSI) in Lenke type 1A curve.

**Methods:**

This study included 106 patients with Lenke Type 1A curve who were followed up more than two years after posterior correction surgery. Pedicle screw (PS) constructs were used in 84 patients, and hybrid constructs in 22. The upper instrumented vertebra was rostral to the upper-end vertebra (UEV) in 70 patients, at UEV in 26, and below UEV in 10. The clavicle angle and T1 tilt angle were measured as PSI indicators, and correlations between radiographic parameters of shoulder balance and other radiographic parameters and associations between PSI and clinical parameters were investigated. For statistical analyses, paired and unpaired t-tests were used.

**Results:**

The mean Cobb angles of the main and proximal thoracic curves were 54.6 ± 9.5 and 26.7 ± 7.9 degrees before surgery, 14.5 ± 7.5, and 14.9 ± 7.1 at follow-up. Clavicle angle and T1 tilt angle were −2.9 ± 2.8 and −2.6 ± 6.3 before surgery, 2.4 ± 2.8 and 4.4 ± 4.3 immediately after surgery, and 1.8 ± 2.1 and 3.4 ± 5.5 at follow-up. Twenty patients developed distal adding-on. Clavicle angle at follow-up correlated weakly but significantly with preoperative clavicle angle (r = 0.34, p = 0.001) and with the correction rates of the main thoracic curve (r = 0.34, p = 0.001); it correlated negatively with the proximal curve spontaneous correction rate (r = −0.21, p = 0.034). The clavicle angle at follow-up was significantly larger in patients with PS-only constructs (PS 2.1 degrees vs. hybrid 0.9, p = 0.02), and tended to be smaller in patients with distal adding-on (adding-on 1.1 vs. non adding-on 2.0, p = 0.09).

**Conclusions:**

PSI was more common with better correction of the main curve (using PS constructs), in patients with a larger preoperative clavicle angle, and with a larger and more rigid proximal curve. Distal adding-on may compensate for PSI.

**Electronic supplementary material:**

The online version of this article (doi:10.1186/1471-2474-15-366) contains supplementary material, which is available to authorized users.

## Background

Adolescent idiopathic scoliosis, of which etiology remains to be clarified [1], is classified into six types by Lenke et al. [2]. Lenke type 1 curve is a single thoracic curve with non-structural flexible curves in the proximal thoracic and lumbar spine. Lenke type 1 has three modifiers—A, B, and C—that indicate the configuration and magnitude of the distal lumbar curve. For Lenke type 1 curve, especially 1A, selective fusion of the main thoracic curve usually has good radiological and clinical outcomes [3], while in Lenke type 1B and 1C, which have more lumbar involvement, the appropriate distal fusion level is often more difficult to determine [4–7].

Recent advancements in surgical techniques to treat adolescent idiopathic scoliosis (AIS), such as the use of pedicle screw (PS) constructs, allow good correction of the main thoracic curve [8–11]. However, maximal correction of the main thoracic curve can cause the left shoulder to elevate, even in Lenke type 1 curves, because a proximal thoracic curve often has enough rigidity to prevent a spontaneous correction equivalent to correction achieved by instrumented fusion in the main thoracic curve [12]. If postoperative shoulder imbalance (PSI) persists, the left shoulder becomes quite prominent, and this may cause patients to be dissatisfied with the results of the surgery [12–14].

Although several factors might influence the onset of post-surgical PSI in Lenke type 1 curve, few studies have examined these factors. This multicenter study was conducted to investigate the occurrence and related factors of PSI in Lenke type 1A curve specifically, since the appropriate distal fusion level is less arguable in Lenke type 1A than in type 1B or 1C.

## Methods

Six scoliosis centers participated in this multicenter study. Approval for this study was obtained from the institutional review board of each participating hospital (Institutional Review Boards of Keio University, Meijo Hospital, Kobe Medical Center, Hokkaido University, Fukuoka Children’s Hospital, and Seirei Citizen Hospital). All patients or their guardians comprehensively consent to participate in the study conducted retrospectively by this study group. The study included 106 patients with Lenke Type 1A curve who underwent posterior correction surgery selectively for the main thoracic curve, and who were followed for 2 years or more (8 males, 98 females; mean age at surgery, 16.2 ± 3.0 years). Each participating center used its own surgical strategy in the present study. All-PS constructs were used in 84 patients, and hybrid constructs using PSs, hooks, and wires were used in 22 patients. The upper instrumented vertebra (UIV) was T2 in one patient, T3 in 21, T4 in 46, T5 in 20, T6 in 15, and T7 in 3. The UIV was rostral to the upper end vertebra (UEV) in 70 patients, at the UEV in 26, and below the UEV in 10. The lower instrumented vertebra (LIV) was T12 in 22 patients, L1 in 51, L2 in 30, and L3 in 3. The LIV was proximal to the lower end vertebra (LEV) in one patient, at the LEV in 55 patients, and distal to the LEV in 50. The mean surgical time was 247.4 ± 66.8 minutes, and the mean estimated blood loss was 829.7 ± 553.2 ml.

### Evaluations

**Figure 1:**
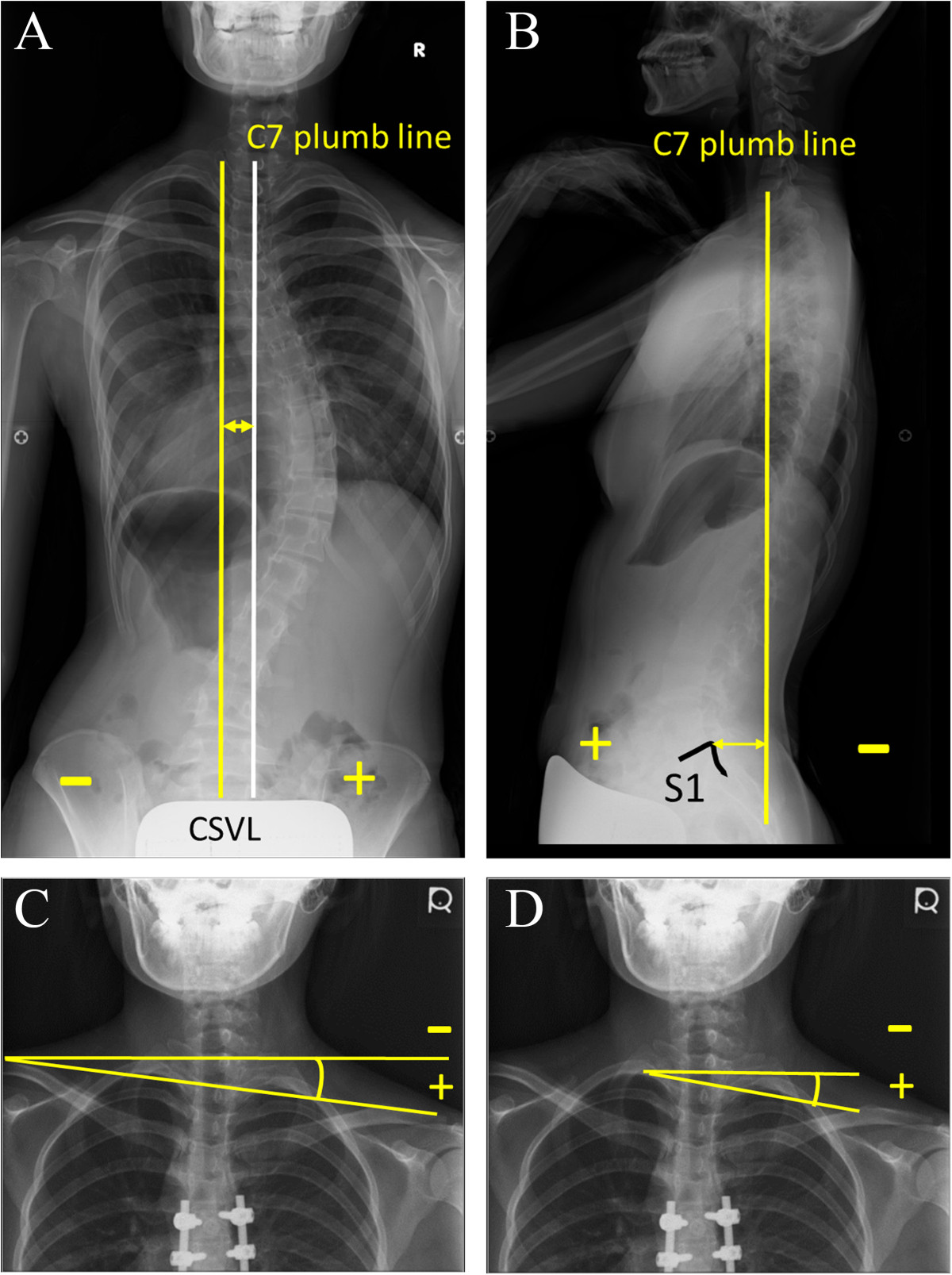
**Radiographic measurements. A**. Coronal balance; a distance between the central sacral vertical line and the C7 plumb line (right; positive, left; negative) in the standing postero-anterior radiograph. **B**. Sagittal balance: a distance between the C7 plumb line and the posterosuperior corner of the sacrum in the lateral standing radiograph. (anterior; positive, posterior; negative). **C**. Clavicle angle: the angulation of a horizontal line and the tangential line connecting the highest two points of each clavicle (left side up; positive, left side down; negative). **D**. T1 tilt angle; the angulation of the upper endplate of T1 to the horizontal line (left side up; positive, left side down; negative).

Each patient’s radiographic data were analyzed for thoracic kyphosis (T5-T12), apical translation of the main thoracic curve, coronal and sagittal balances, and the Cobb angles of the lumbar curve and the main and proximal thoracic curves [15]. Side benders, right and left side bending films taken in the supine position, were taken and the flexibility was calculated as (the Cobb angle in the standing film-that in the side bender)/the Cobb angle in the standing film × 100 (%). The correction rate was calculated as (the preoperative Cobb angle – the postoperative or the follow-up Cobb angle)/the preoperative Cobb angle in the standing film × 100 (%). The coronal balance was defined as a distance between the central sacral vertical line and the C7 plumb line (right; positive, left; negative) in the standing postero-anterior radiograph, and the sagittal balance as a distance between the C7 plumb line and the posterosuperior corner of the sacrum in the lateral standing radiograph (Figure 1) [15]. The clavicle angle and T1 tilt angle were measured and used to indicate shoulder balance [15]. The clavicle angle was defined by the angulation of a horizontal line and the tangential line connecting the highest two points of each clavicle; the T1 tilt angle was defined as the angulation of the upper endplate of T1 to the horizontal line. A clavicle angle and T1 tilt angle were positive when the left side was raised (Figure 1).

Distal adding-on was defined according to Wang et al. as a progressive increase in the number of vertebrae included within the distal curve, with either an increase of more than 5 mm in the deviation of the first vertebra below the instrumentation from the CSVL (the vertical line bisecting the proximal sacrum), or an increase of more than 5 degrees in the angulation of the first disc below the instrumentation [16]. All measurements were obtained from digital data from standing posterior-anterior and lateral long cassette films taken before, immediately after, and 2 years after surgery. CIS-Image/Viewer software ver. 2.11.31 (IBM Japan, Tokyo, Japan) was used to obtain the measurements. All measurements were conducted by one of the authors (KW) who is an experienced scoliosis surgeon. Clinical outcomes were evaluated from SRS 22 scores at follow-up.

### Statistical analyses

Paired t-tests were used to compare radiographic parameters before surgery and at the 2-year follow-up; unpaired t-tests or ANOVA were used to compare continuous variables such as radiographic parameters between two or three groups; and Pearson’s correlation coefficients were used to assess the linear correlation between radiographic parameters. A statistical software package, IBM SPSS Statistics 20 (IBM Japan, Tokyo), was used for the analyses, and a p-value of less than 0.05 was considered statistically significant.

## Results

### Radiographic evaluation

The mean Cobb angles of the main and proximal thoracic curves and the lumbar curve were 54.6 ± 9.5, 26.7 ± 7.9, and 29.1 ± 5.8 degrees before surgery and 14.5 ± 7.5, 14.9 ± 7.1, and 6.2 ± 4.5 at follow-up. The correction rates were 73.5 ± 13.0%, 44.3 ± 22.6, and 79.2 ± 14.5 respectively. Thus, the three curves, but especially the main thoracic curve and the lumbar curve, improved significantly after surgery (p = 0.001) (Table 1). The correction rate of the main thoracic curve was 75.4 ± 11.3% in patients treated with all-PS constructs, and 66.4 ± 16.2% in those treated with hybrid constructs (p = 0.003).

**Table 1:** **Radiographic data**

	Preop.	Immediately postop	Follow-up 2 years
**Cobb angle**			
**Main thoracic curve**	**54.6 ± 9.5 (52.8-56.5)**	**13.4 ± 7.0 (12.1-14.8)**	**14.5 ± 7.5 (13.1-16.0)**
Side bender (°)	28.8 ± 11.6 (26.6-31.0)		
Flexibility (%)	47.8 ± 17.4 (44.4-51.1)		
Correction rate (%)		75.7 ± 11.4 (73.5-77.9)	73.5 ± 13.0 (71.0-76.0)
**Proximal thoracic curve**	**26.7 ± 7.9 (25.1-28.2)**	**14.9 ± 6.4 (13.7-16.2)**	**14.9 ± 7.1 (13.5-16.3)**
Side bender (°)	17.9 ± 6.7 (16.5-19.2)		
Flexibility (%)	34.5 ± 28.4 (29.0-40.0)		
Correction rate (%)		44.0 ± 18.3 (40.5-47.5)	44.3 ± 22.6 (39.9-48.6)
**Lumbar curve**	**29.1 ± 5.8 (28.0-30.2)**	**6.2 ± 4.7 (5.3-7.1)**	**6.2 ± 4.5 (5.3-7.1)**
Side bender (°)	2.5 ± 8.7 (0.8-4.2)		
Flexibility (%)	93.3 ± 31.3 (87.2-99.3)		
Correction rate (%)		79.2 ± 14.3 (76.5-82.0)	79.2 ± 14.5 (76.4-82.0)
Thoracic kyphosis (T5-12) (°)	12.6 ± 10.7 (10.5-14.6)	13.6 ± 7.4 (12.2-15.0)	15.7 ± 13.7 (13.1-18.4)
Coronal balance (mm)	**6.6 ± 14.9 (3.7-9.5)**	**−2.5 ± 12.1 (−4.8- -0.2)**	**−1.2 ± 10.8 (−3.3-0.8)**
Sagittal balance (mm)	**−4.9 ± 25.6 (−9.9- -0.1)**	**4.2 ± 31.7 (−2.0-10.5)**	**−16.1 ± 24.6 (−20.8- -11.3)**
Apical translation (mm)	**53.7 ± 20.7 (49.7-57.7)**	**15.0 ± 12.2 (12.2-17.4)**	**16.6 ± 12.9 (14.1-19.1)**
Clavicle angle (°)	**−2.9 ± 2.8 (−3.4- -2.3)**	**2.4 ± 2.8 (1.9-2.9)**	**1.8 ± 2.1 (1.4-2.2)**
T1 tilt angle (°)	**−2.6 ± 6.3 (−3.4- -2.3)**	**4.4 ± 4.3 (3.5-5.2)**	**3.4 ± 5.5 (2.3-4.4)**

The numbers in the parentheses indicate 95% confidence interval.

Bold letters indicate statistical significance by paired t-tests.

The clavicle angle was −2.9 ± 2.8 degrees before surgery, 2.4 ± 2.8 immediately after surgery, and 1.8 ± 2.1 at follow-up (p = 0.001). The clavicle angle was positive in only 7 patients (6.6%) before surgery, in 88 patients (83%) immediately after surgery, and in 79 patients (74.5%) at follow-up. The T1 tilt angle was −2.6 ± 6.3 degrees before surgery, 4.4 ± 4.3 immediately after surgery, and 3.4 ± 5.5 at follow-up (p = 0.001). The T1 tilt angle was positive in 33 patients (31.1%) before surgery, in 79 patients (74.5%) immediately after surgery, and in 74 patients (69.8%) at follow-up.

### Correlations between radiographic parameters of shoulder balance and other radiographic parameters

The clavicle angle at follow-up was weakly but significantly correlated with the correction rate of the main thoracic curve (r = 0.34, p = 0.001) and of the apical translation (r = 0.32, p = 0.001), and with the preoperative clavicle angle (r = 0.34, p = 0.001) (Table 2). The clavicle angle at follow-up was negatively correlated with the spontaneous correction rate of the proximal curve (r = −0.21, p = 0.034).

**Table 2:** **Pearson’s correlations between radiographic parameters of shoulder balance and other radiographic parameters**

	Clavicle angle at 2 years		T1 tilt angle at 2 years	
	Correlation Coefficient	p-value	Correlation Coefficient	p-value
Preop. Cobb angle of MT (°)	0.05	0.64	0.13	0.20
Flexibility of MT (%)	0.08	0.39	0.05	0.59
Correction rate of MT (%)	0.34*	0.001	0.12	0.22
Apical translation of MT (mm)	−0.18	0.07	−0.09	0.35
Correction rate of apical translation of MT (%)	0.32*	0.001	0.09	0.39
Preop. Cobb angle of PT (°)	0.12	0.22	0.42*	0.001
Flexibility of PT (%)	0.06	0.55	−0.28*	0.005
Correction rate of PT (%)	−0.21*	0.034	−0.32*	0.001
Preop. Cobb angle of LC (°)	−0.10	0.30	−0.004	0.97
Flexibility of LC (%)	−0.02	0.85	0.17	0.09
Correction rate of LC (%)	0.09	0.34	−0.13	0.20
Preop. clavicle angle (°)	0.34*	0.001	0.13	0.20
Preop. T1 tilt angle (°)	0.15	0.12	0.35*	0.001

MT; main thoracic curve, PT; proximal thoracic curve, LC; lumbar curve.

Asterisks indicate statistical significance.

The T1 tilt angle at follow-up was weakly but significantly correlated with the preoperative T1 tilt angle (r = 0.35, p = 0.001) and with the preoperative Cobb angle of the proximal thoracic curve (r = 0.42, p = 0.001), and negatively correlated with the flexibility of the proximal thoracic curve (r = −0.28, p = 0.005) and its correction rate (r = −0.32, 0.001) (Table 2).

We also compared radiographic parameters between patients with positive clavicle and T1 tilt angles at follow-up (clavicle angle, T1 tilt angle >0°) and those without (Table 3). Correction rate of the main thoracic curve, that of the apical translation of the main thoracic curve, preoperative clavicle angle and T1 tilt angle were significantly larger in patients with positive clavicle angle than those without. In those with positive T1 tilt angle, preoperative Cobb angle and flexibility of the proximal thoracic curve and preoperative T1 tilt angle were significantly larger and correction rate of the proximal thoracic curve was significantly smaller than those without positive T1 tilt angle.

**Table 3:** **Comparison between patients with positive clavicle angle and T1 Tilt angle and those without**

	Clavicle angle at 2 years			T1 tilt angle at 2 years		
	> 0	≤ 0	p-value	> 0	≤ 0	p-value
Preop. Cobb angle of MT (°)	53.9 ± 8.6	56.7 ± 11.6	0.19	55.4 ± 9.7	52.8 ± 9.0	0.20
Flexibility of MT (%)	48.9 ± 16.7	44.6 ± 19.3	0.27	48.41 ± 7.7	46.4 ± 17.0	0.59
Correction rate of MT (%)	75.1 ± 12.0	68.4 ± 14.5	0.02*	74.5 ± 12.7	71.1 ± 13.5	0.22
Apical translation of MT (mm)	50.7 ± 17.3	62.1 ± 27.4	0.01*	52.7 ± 18.7	55.8 ± 24.9	0.48
Correction rate of apical translation of MT (%)	50.7 ± 17.0	62.1 ± 27.4	0.005*	70.7 ± 19.5	67.0 ± 21.0	0.39
Preop. Cobb angle of PT (°)	26.7 ± 8.1	26.4 ± 7.5	0.86	28.8 ± 6.7	21.7 ± 8.2	0.001*
Flexibility of PT (%)	34.4 ± 30.4	34.7 ± 21.5	0.97	29.3 ± 28.0	46.2 ± 25.9	0.005*
Correction rate of PT (%)	41.9 ± 23.8	51.2 ± 17.4	0.07	39.6 ± 17.8	55.0 ± 28.5	0.001*
Preop. Cobb angle of LC (°)	28.7 ± 5.3	30.2 ± 6.9	0.25	29.1 ± 5.8	29.1 ± 5.7	0.97
Flexibility of LC (%)	92.5 ± 30.8	95.5 ± 33.3	0.67	96.7 ± 33.2	85.5 ± 251	0.09
Correction rate of LC (%)	80.4 ± 4.2	75.5 ± 15.4	0.16	80.4 ± 13.5	76.5 ± 16.6	0.20
Preop. clavicle angle (°)	−2.3 ± 2.4	−4.5 ± 3.2	0.001*	−2.6 ± 2.7	−3.4 ± 3.0	0.20
Preop. T1 tilt angle (°)	−1.6 ± 5.9	−5.3 ± 8.8	0.017*	−1.0 ± 6.6	−6.3 ± 6.4	0.001*

MT; main thoracic curve, PT; proximal thoracic curve, LC; lumbar curve.

Asterisks indicate statistical significance.

#### Relationships between radiographic parameters of shoulder balance and clinical factors

The clavicle angle was significantly larger in patients treated with PS-only constructs than in those treated with hybrid constructs (PS 2.1 degrees vs. hybrid 0.9, p = 0.02), and tended to be smaller in patients with distal adding-on than in those without (adding-on 1.1 vs. non adding-on 2.0, p = 0.09) (Table 4).

**Table 4:** **Relationships between radiographic parameters of shoulder balance and clinical factors**

	N	Clavicle angle at 2 years	p-value (95% confidence interval)	T1 tilt angle at 2 years	p-value (95% confidence interval)
Implant					
Hybrid	22	0.87 ± 2.21	0.019* (−0.11-1.85)	1.22 ± 4.69	0.04* (−0.85-3.31)
All Pedicle screw	84	2.05 ± 2.04	(1.61-2.49)	3.95 ± 5.63	(2.72-5.17)
UIV					
Rostral to EV	70	2.19 ± 1.83	0.56 (1.15-2.22)	4.22 ± 5.29	0.047* (2.95-5.48)
EV	26	1.69 ± 2.23	(1.45-2.93)	2.43 ± 5.74	(0.12-4.75)
Caudal to EV	10	1.69 ± 2.02	(0.16-3.06)	0.01 ± 5.59	(−3.99- 4.01)
Distal Adding On					
+	20	1.07 ± 2.37	0.086 (−0.09- 2.23)	2.86 ± 5.16	0.045* (1.76-3.97)
-	86	1.97 ± 2.01	(1.54-2.41)	5.62 ± 6.63	(2.51-8.72)
Risser grade					
≥ 4	82	1.87 ± 2.22	0.56 (1.38-2.35)	3.78 ± 5.72	0.33 (2.52-5.04)
<4	23	1.55 ± 1.80	(0.78-2.32)	2.26 ± 4.70	(0.22-4.29)

UIV; Upper instrumented vertebra, EV; end vertebra.

*Statistically significant.

The T1 tilt angle at follow-up was significantly larger in patients with a UIV rostral to the end vertebra (p = 0.047), while the clavicle angle was not different among patients with a UIV rostral to, and or caudal to the end vertebra (p = 0.56), and in patients treated with PS-only as compared to hybrid constructs (PS 4.0 degrees vs. hybrid 1.2, p = 0.04); it was smaller in patients with distal adding-on than those without (adding-on; 2.9 vs. non adding-on; 5.6, p = 0.045) (Table 4).

### Clinical evaluation

SRS 22 scores at follow-up are shown in Table 5. There was no significant correlation between the total SRS 22 score or that of any domain and the clavicle or T1 tilt angle.

**Table 5:** **Shoulder balance and SRS outcome scores**

	Mean ± SD	Clavicle angle at 2 years		T1 tilt angle at 2 years	
		Correlation coefficient	p-value	Correlation coefficient	p-value
SRS 22 scores at follow-up					
Function	4.4 ± 0.5	−0.02	0.88	−0.22	0.13
Pain	4.5 ± 0.4	−0.08	0.60	0.14	0.35
Self image	3.8 ± 0.6	−0.15	0.31	0.02	0.87
Mental health	4.1 ± 0.7	−0.13	0.38	−0.23	0.11
Satisfaction	4.0 ± 0.7	−0.10	0.52	−0.10	0.49
Total	4.2 ± 0.4	−0.14	0.36	−0.14	0.33

## Discussion

In the present study, the proximal thoracic curve improved from 26.7 degrees before surgery to 14.9 degrees at follow-up (correction rate 44.3%), but the proximal thoracic curve correction rate was worse than the rates for the main thoracic and lumbar curves.

In patients with Lenke type 1 curve, the proximal thoracic curve may correct spontaneously when the main thoracic curve is corrected surgically [17–19]. Kuklo et al. found that spontaneous correction of the proximal thoracic curve occurred consistently after selective, instrumented fusion of the main thoracic curve in 44 patients treated by posterior surgery and 41 treated by anterior surgery [17]; the preoperative flexibility of the proximal curve correlated positively with spontaneous correction. In a study of the surgical results of 40 patients treated by anterior corrective surgery, Lee et al. found that patients with mild left shoulder elevation could be treated by anterior correction if the magnitude of the proximal thoracic curve was less than 30 degrees [19].

In this study, the mean clavicle angle and T1 tilt angle were positive in 75% and 70% of the patients at follow-up, respectively, indicating that some degree of PSI developed frequently in patients treated surgically for Lenke type 1A curve. PSI was more common in patients with better correction of the main curve, which was achieved using PS constructs. PSI was also more common in patients with a larger preoperative clavicle or T1 tilt angle, a larger and more rigid proximal curve, larger correction rate of the main thoracic curve and those with a lesser degree of spontaneous proximal thoracic curve correction observed at follow-up.

The level of upper instrumented vertebra was significantly correlated with the T1 tilt angle but not with the clavicle angle. The T1 tilt angle was significantly larger when the upper instrumented vertebra was rostral to UEV. Because most of the upper end vertebra was at T3 or below, the somewhat structural proximal thoracic curve might not be well controlled by instrumentation. The clavicle angle might not be so directly influenced by the proximal thoracic curve as the T1 tilt angle.

We found no significant correlation between the SRS 22 scores and radiographic parameters of shoulder balance. This may be because most patients obtained good correction of the main thoracic curve, and the PSI was not severe enough to impact the patients’ satisfaction with the results of the surgery. PS constructs allow maximum correction of the main thoracic curve, which can, paradoxically, cause shoulder imbalance. This results from the proximal thoracic curve, which is often somewhat inflexible, not bending out in response to the correction of the main thoracic curve, causing the left shoulder to elevate after surgery.

Our results partly correspond to results reported by previous studies. Kuklo et al. evaluated PSI in 112 patients with AIS by dividing them into four groups based on the extent of inclusion of the proximal thoracic curve into the instrumented fusion and on the surgical approach (posterior or anterior) [20]. They achieved good postoperative shoulder balance in each group, and found that the preoperative clavicle angle correlated with postoperative shoulder balance. Clinical appearance was improved, and the overall postoperative SRS 22 scores were acceptable.

**Figure 2:**
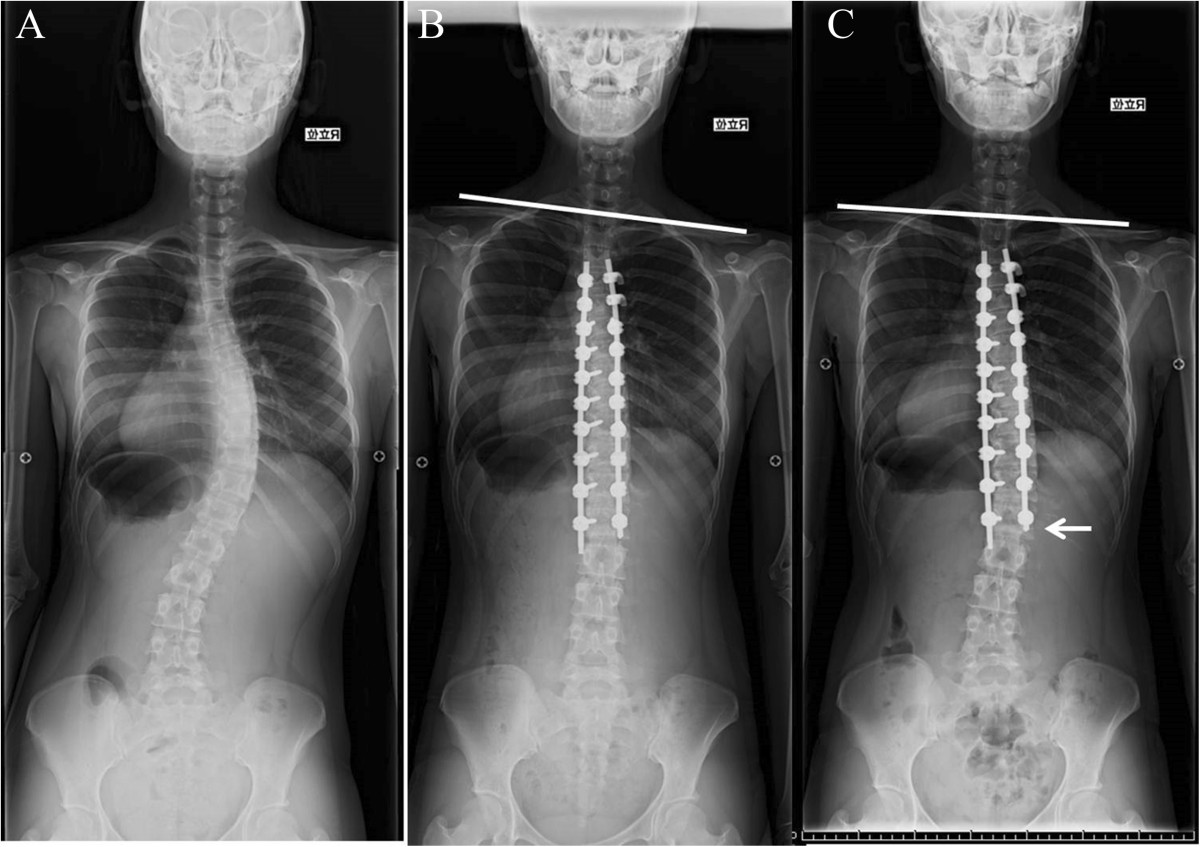
**A 17-year-old female who underwent posterior correction and fusion using PS constructs.** She developed PSI with good correction of the main thoracic curve. The PSI improved at follow-up, as distal adding-on developed (white arrow). **A**. Before surgery, **B**. Immediately after surgery, **C**. At the two-year follow-up.

PSI may be compensated for by the development of distal adding-on (Figure 2). As yet, it is not known whether distal adding-on develops independently or correlatively to PSI to rebalance the left shoulder elevation. If correlatively, surgeons should do their best to prevent PSI, since distal adding-on can eventually result in symptomatic degenerative changes of the lumbar spine [21]. Several surgical options to prevent PSI have been reported, including extending the fusion levels to the rostral vertebrae (such as T1 and T2) and tempering the correction of the main curve by setting the UIV below the UEV (short fusion strategy, as reported by Matsumoto et al. [22]), or by limiting the correction obtained within the instrumented vertebrae [12].

There are several limitations to the present study. First, although this multicenter study has a scale merit, the determination of UIV and LIV, the type of instrumentation used, and the surgical techniques used varied depending on each participating facility; these may be confounding factors. Second, this study focuses only on a specific curve type, Lenke type 1A, to eliminate a possible confounding factor (i.e., lumbar modifiers), and it remains to be clarified whether the results obtained are applicable to other types of AIS.

## Conclusion

In conclusions, this study clarified factors significantly related to the onset of PSI. PSI was more common with better correction of the main curve (using PS constructs), in patients with a larger preoperative clavicle angle, and with a larger and more rigid proximal thoracic curve. To prevent PSI for Lenke type 1A curve patients with these factors, maximum correction of the proximal thoracic curve with the upper instrumented vertebra at T2 or above or limited correction of the main thoracic curve should be considered.

## Authors’ original submitted files for images

Below are the links to the authors’ original submitted files for images. Authors’ original file for figure 1 Authors’ original file for figure 2 Authors’ original file for figure 3 Authors’ original file for figure 4 Authors’ original file for figure 5
