# Supplementary figures and images for: Postoperative shoulder imbalance in Lenke Type 1A adolescent idiopathic scoliosis and related factors
Source: BMC Musculoskelet Disord. 2014 Nov 5;15:366. doi: 10.1186/1471-2474-15-366 (PMC4230354; doi:10.1186/1471-2474-15-366)

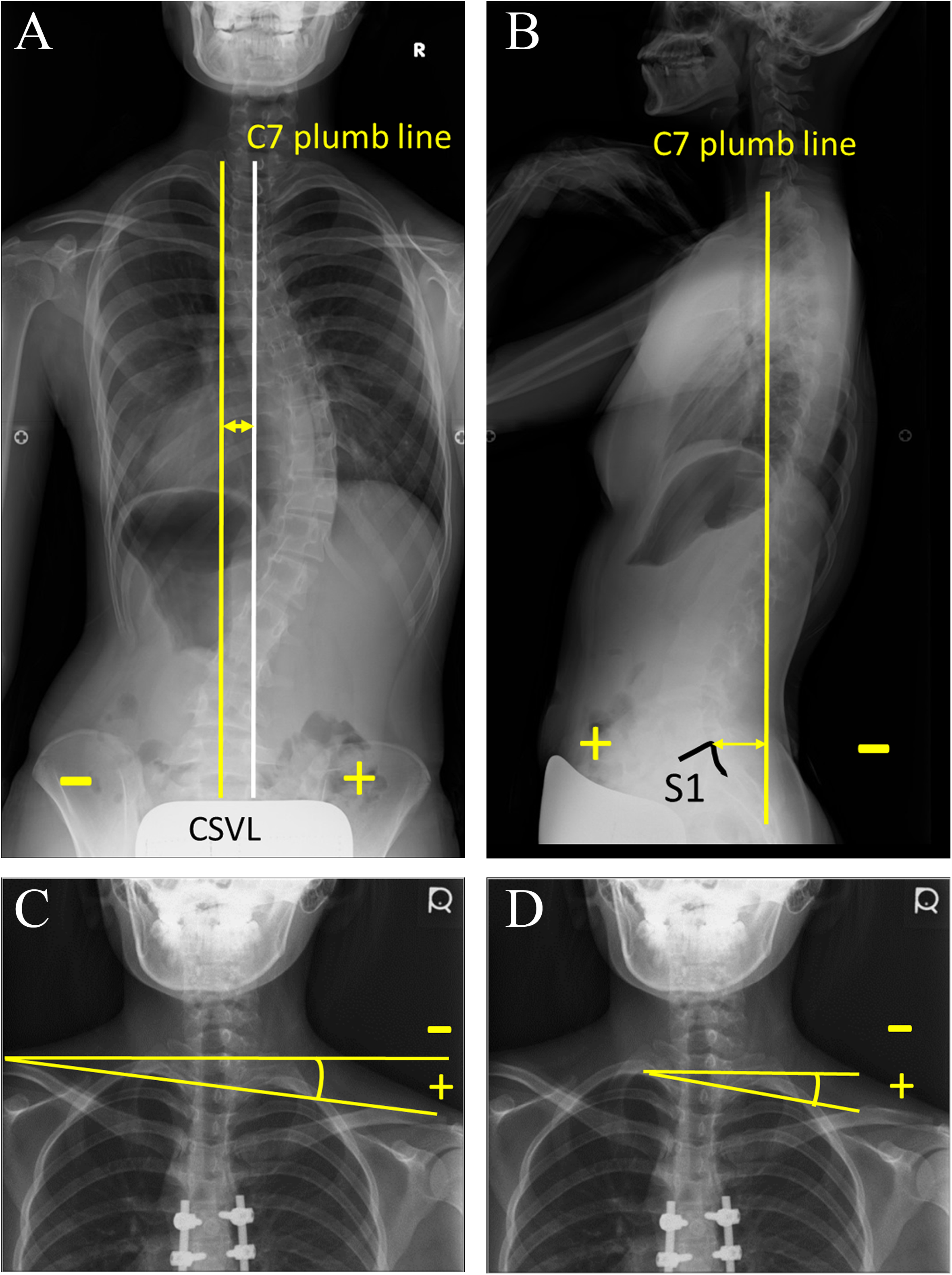

Supplement: Supplementary file 1 — Authors’ original file for figure 1 [file 12891_2014_2301_MOESM1_ESM.tif]

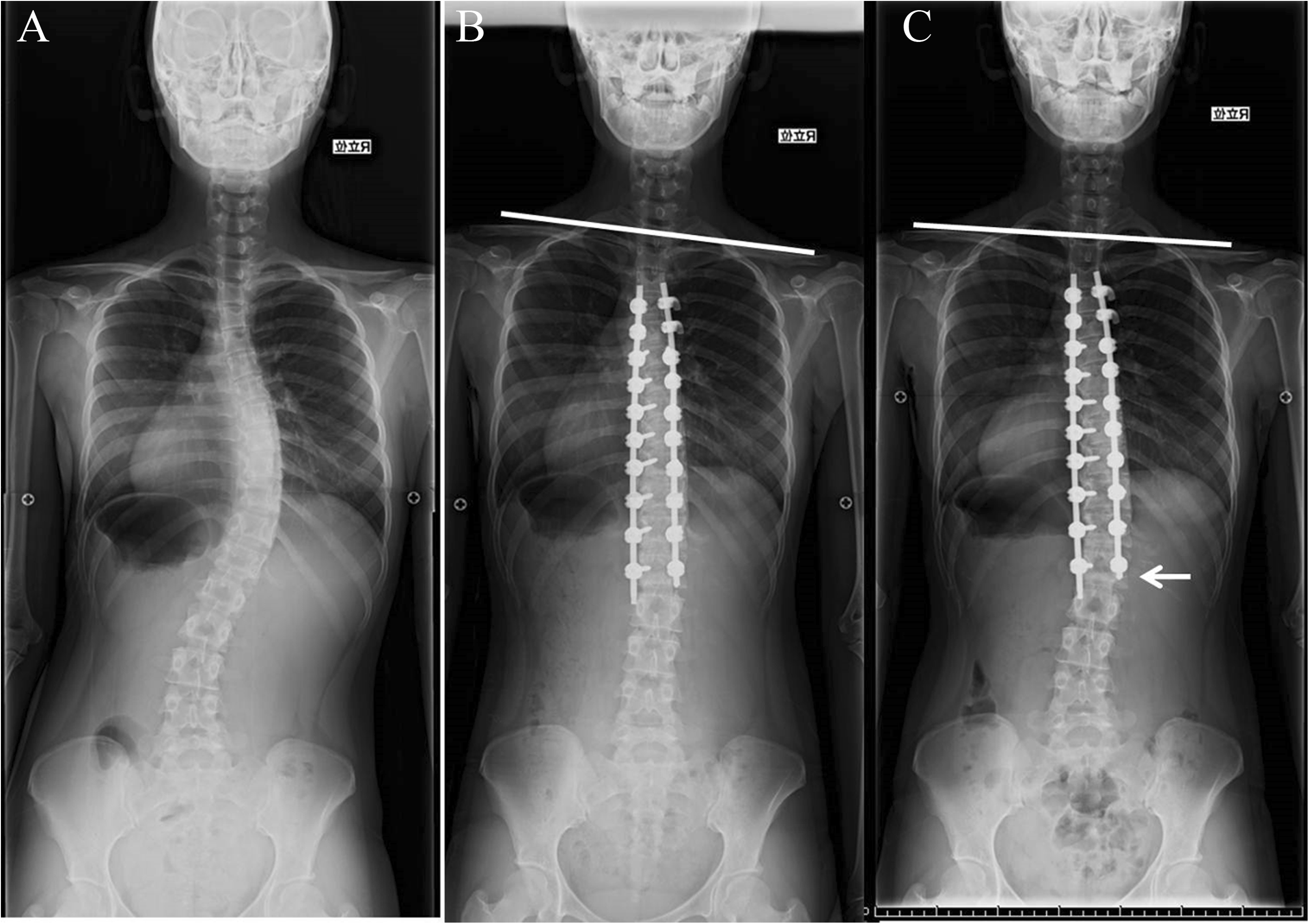

Supplement: Supplementary file 2 — Authors’ original file for figure 2 [file 12891_2014_2301_MOESM2_ESM.tiff]

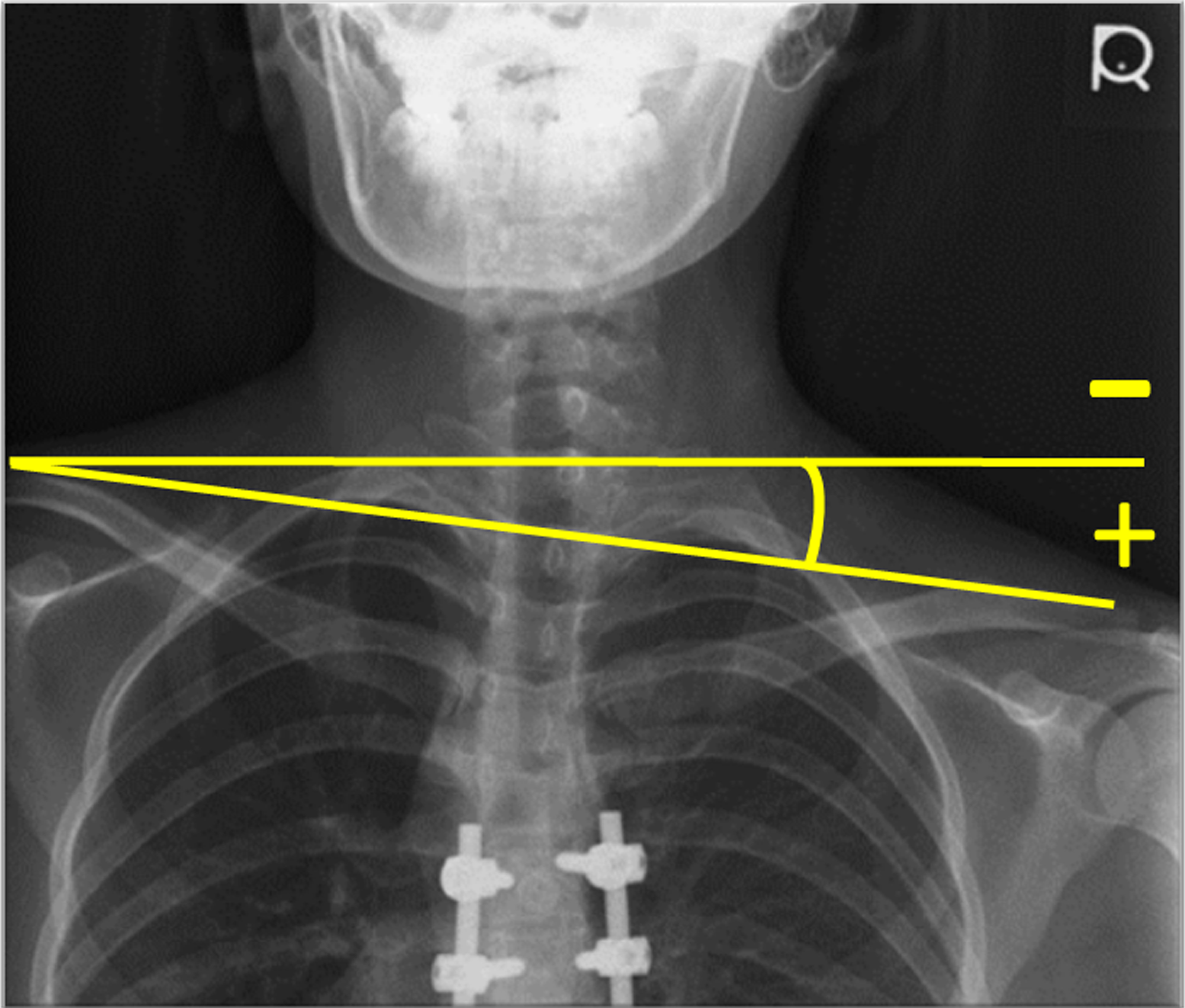

Supplement: Supplementary file 3 — Authors’ original file for figure 3 [file 12891_2014_2301_MOESM3_ESM.tiff]

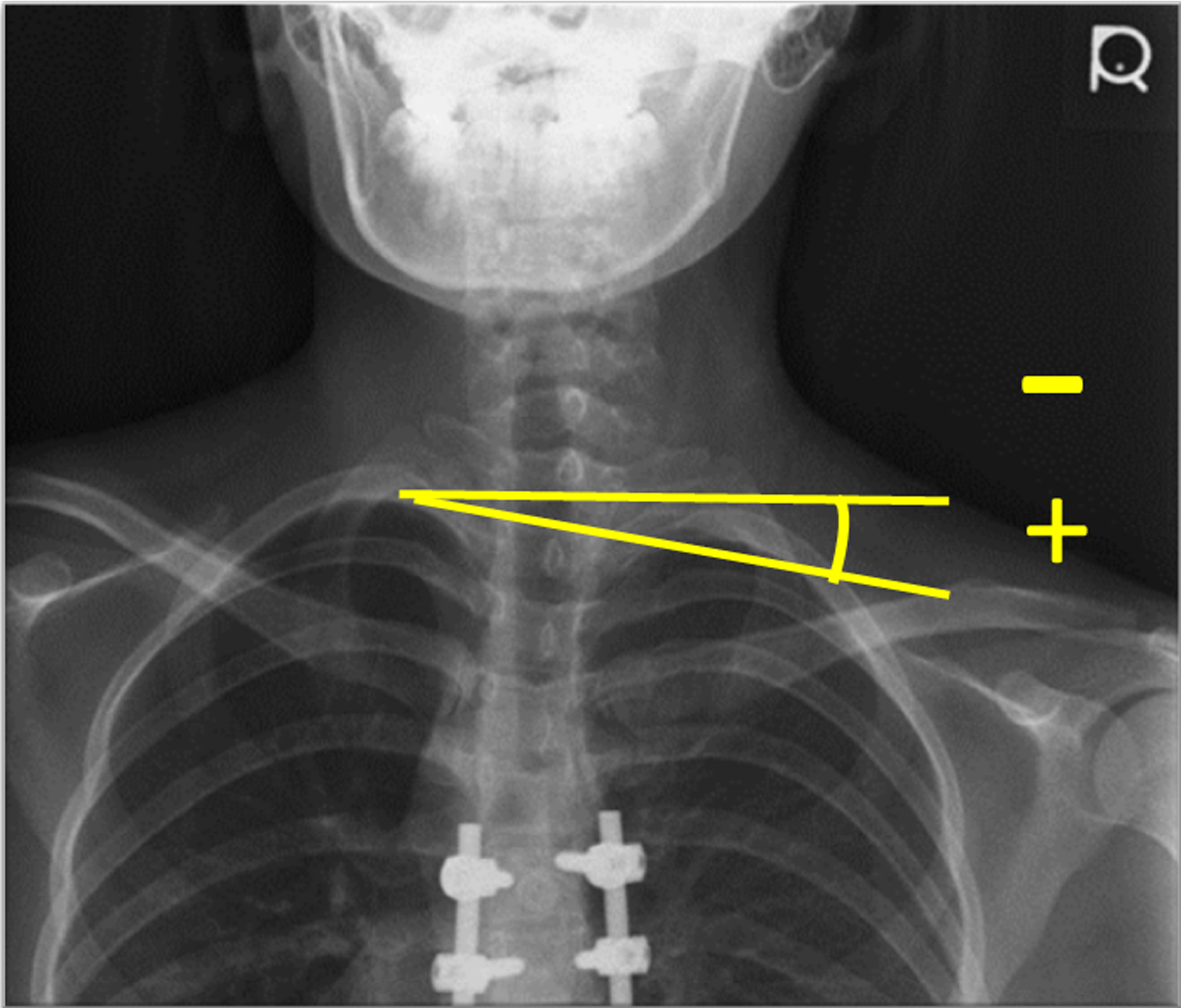

Supplement: Supplementary file 4 — Authors’ original file for figure 4 [file 12891_2014_2301_MOESM4_ESM.tiff]

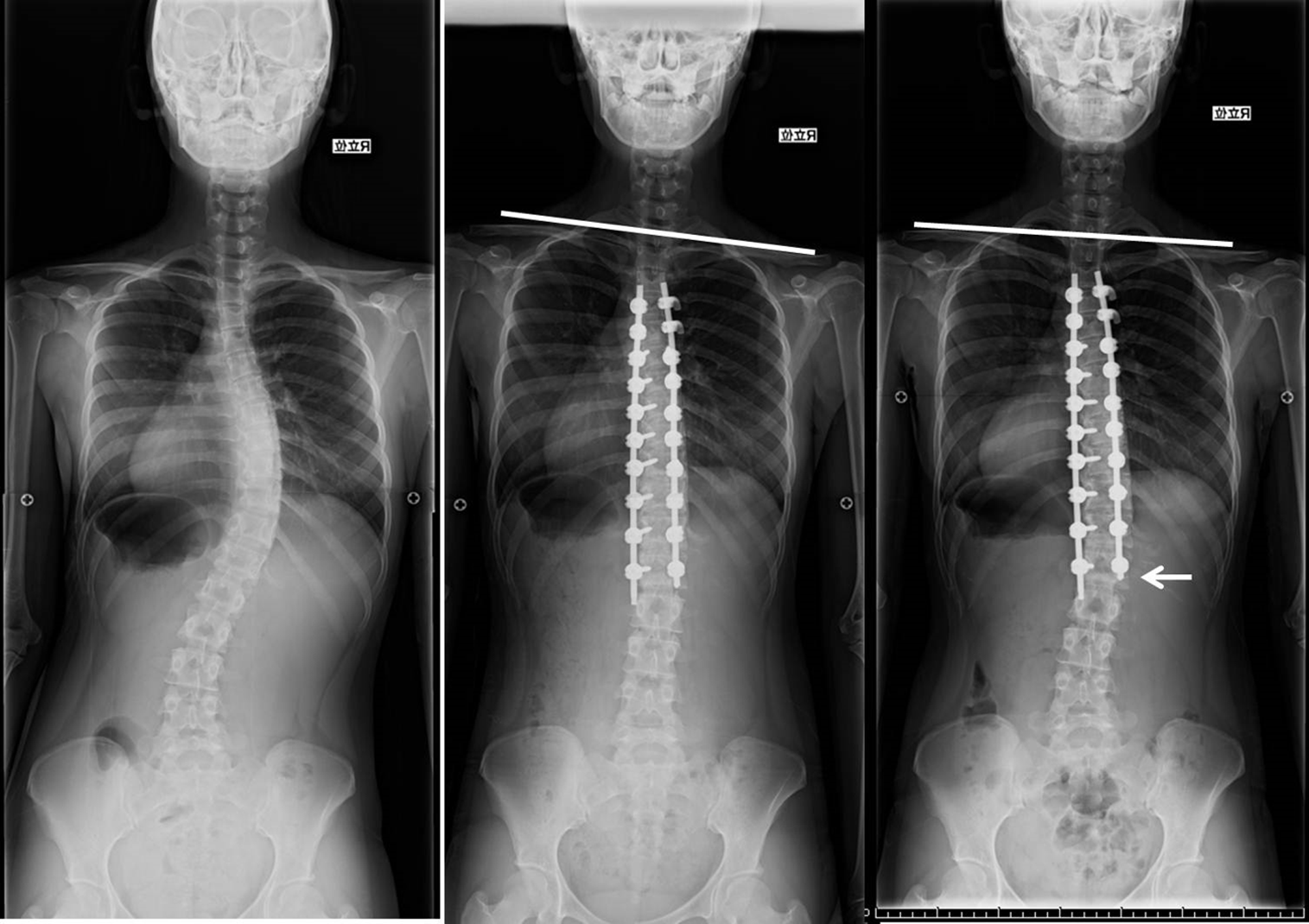

Supplement: Supplementary file 5 — Authors’ original file for figure 5 [file 12891_2014_2301_MOESM5_ESM.tiff]
